# Promoting physical activity with a school-based dance mat exergaming intervention: qualitative findings from a natural experiment

**DOI:** 10.1186/s12889-016-3308-2

**Published:** 2016-07-20

**Authors:** Duika Burges Watson, Jean Adams, Liane B. Azevedo, Catherine Haighton

**Affiliations:** School of Medicine, Pharmacy and Health, Durham University, Stockton-on-Tees, UK; Institute of Health & Society, Newcastle University, Baddiley-Clark Building, Richardson Road, Newcastle upon Tyne, NE2 4AX UK; School of Health and Social Care, Teesside University, Middlesbrough, UK; MRC Epidemiology Unit and UKCRC Centre for Diet and Activity Research (CEDAR), University of Cambridge School of Clinical Medicine, Cambridge, UK

**Keywords:** Physical activity, Children, Sociology of translation, Qualitative, Exergaming

## Abstract

**Background:**

Physical activity is critical to improving health and well-being in children. Quantitative studies have found a decline in activity in the transition from primary to secondary education. Exergames (active video games) might increase physical activity in adolescents. In January 2011 exergame dance mat systems were introduced in to all secondary schools across two local authority districts in the UK. We performed a quasi-experimental evaluation of a natural experiment using a mixed methods design. The quantitative findings from this work have been previously published. The aim of this linked qualitative study was to explore the implementation of the dance mat scheme and offer insights into its uptake as a physical activity intervention.

**Methods:**

Embedded qualitative interviews at baseline and 12 month follow-up with purposively selected physical education teachers (*n* = 20) and 25 focus groups with a convenience sample of pupils (*n* = 120) from five intervention schools were conducted. Analysis was informed by sociology of translation approach.

**Results:**

At baseline, participants (both teachers and pupils) reported different expectations about the dance mats and how they could be employed. Variation in use was seen at follow-up. In some settings they were frequently used to engage hard to reach groups of pupils. Overall, the dance mats were not used routinely to increase physical activity. However there were other unanticipated benefits to pupils such as improved reaction time, co-ordination and mathematic skills. The use of dance mats was limited in routine physical education classes because of contextual issues (school/government policy) technological failures (batteries/updates) and because of expectations about how and where they could be used.

**Conclusions:**

Our linked quantitative study (previously published) suggested that the dance mats were not particularly effective in increasing physical activity, but the qualitative results (reported here) show that the dance mats were not used routinely enough to show a significant effect on physical activity of the intervention. This research demonstrates the benefit of using mixed methods to evaluate complex physical activity interventions. Those planning any intervention for promoting physical activity in schools need to understand the distinction between physical activity and physical education.

**Electronic supplementary material:**

The online version of this article (doi:10.1186/s12889-016-3308-2) contains supplementary material, which is available to authorized users.

## Background

Adolescence, the years from puberty to adulthood, is important in the development of lifelong behaviours including regular physical activity (PA) [1]. Increasing PA is a priority to improve health and well-being, however there is a marked decline in activity in the transition (around the age of 11 years) from primary to secondary school [2, 3]. Changes in both social and physical environments during the transition from primary to secondary school appear to be important determinants of the decline in PA seen at this time, with reduced opportunities and social support to be physically active seen alongside increased worry about competition and competence [4]. A systematic review of interventions to promote PA in children and adolescents found that multi-component school-based activities that were gender-sensitive and peer supported were the most promising [5]. However, the highest quality studies in this review were mostly from the US and culturally appropriate interventions elsewhere are under-explored. Another review identified that interventions that were computer-tailored and empowered members of the school community were most effective at engaging adolescents in physical activity [6].

One such multi-component activity that could be gender-sensitive, peer-supported and may increase PA in adolescents is exergaming. Exergames are active video games which include dancing, balance board simulators and virtual sports simulators. There is evidence to support the role of exergames in promoting positive attitudes to PA and improving physical activity self-efficacy in children [7]. Likewise, exergames show promise as a means to increase PA levels [8, 9] including in inactive children during physical education (PE) lessons [10]. However, the majority of studies have focused on short term PA outcomes [8, 11] with limited evidence on the long term effectiveness of exergames [12, 13]. Some studies have specifically addressed gender concerns however there is little research addressing the broader issues of culture [5].

In January 2011 exergame dance mat systems were introduced in to all secondary schools (*n* = 22) across two local authority districts in the UK. Dance mats are a combination of computer game and PA in which dance steps are projected onto a wall or screen and players follow them on foot-activated floor pads. The dance mat scheme was a regional initiative of local National Health Service (NHS) and local authority partners. The secondary schools were each provided with a 32 mat system that could be used independently by individual schools or wirelessly linked to enable interschool and external competition. The dance mat scheme was overseen by a multi-agency steering group of stakeholders, including school sports leads and public health officials. This steering group aimed to encourage greater PA amongst secondary school students; and facilitate wider use of the dance mats in local primary schools, through intercollegiate competitions and during after school programmes. Schools’ agreement with the local authority involved a commitment to use the mats for an initial 6 week pilot. Mats belonged to schools and there was encouragement from the steering group to continue to use the dance mats beyond this pilot phase in scheduled PE classes, breaks, lunchtime, and outside of school hours. The company that supplied the mats were contracted to provide initial training on their use, and ongoing technical support and updates (system, music).

As the dance mat scheme was limited to two local authority districts, we had the opportunity to evaluate a ‘natural experiment’ of the effects of a large scale exergame intervention over a long period, in a ‘real-world’ setting. By recruiting schools from neighbouring local authority districts, that were not taking part in the scheme, we were able to conduct a quasi-experimental evaluation of the effects of the intervention on quality of life domains, body mass index (BMI) and PA in 11–13 year old children over 12 months. Our quantitative findings, presented elsewhere [14] (Additional file 1), were that the intervention was associated with improvement in the quality of life domains psychological well-being, autonomy, parent relations, as well as a decrease in BMI [14]. However, there was a negative effect on PA overall.

Alongside the quantitative evaluation of the dance mats scheme [14], we also conducted a qualitative evaluation. Qualitative research can be useful in teasing out the contextual, social and cultural aspects important to longer-term effectiveness of public health interventions [15]. Indeed, it has been proposed that qualitative approaches are fundamental to the development and evaluation of complex public health interventions [16]. However, qualitative methods are not commonly used to capture contextual information about school-based PA promotion interventions. Some mixed methods studies use qualitative methods to explore the acceptability of exergame interventions, others have considered the broader contextual issues in relation to PA for non-exergame related interventions [17, 18], but we are not aware of any studies that have used qualitative methods to explore the broader cultural and social context of exergame interventions in schools.

The aim of this qualitative study was to explore and understand the successful and unsuccessful elements of the dance mats scheme in order to help explain and interpret the findings from the quantitative evaluation [14]. In order to provide a strong theoretical framework [16] we drew on elements of a sociology of translation (see below) to take into account expectations of users, and ways in which the technologies were integrated (or not) in the already complex settings in which they were placed [19, 20].

## Methods

We conducted a qualitative study with PE teachers and pupils from the five intervention schools that participated in our quantitative evaluation [14]. Teachers took part in individual interviews and pupils in focus groups. Data was collected at both baseline, before intervention implementation, and at 12 month follow up.

### Participants

A total of 20 teachers based at intervention schools participated in in-depth interviews with the lead qualitative researcher (Duika Burges Watson PhD) at baseline, before intervention implementation. Included teachers were aware of the dance mat scheme and its evaluation and were available for interview. Teachers were purposively selected for inclusion by their managers based on meeting the criteria listed above.

Twelve months after implementation, eight of the original 20 teachers were no longer in employment with the same schools (most had originally been employed under a government scheme that was subject to substantial cuts during the evaluation period). Therefore 12 month follow up interviews were undertaken with the remaining 12 teachers.

Pupils were invited by their PE teacher to volunteer for focus group sessions during their regular PE sessions. We conducted focus groups at baseline, before intervention implementation and at 12 months follow up. This convenience sample of pupils was therefore not necessarily the same at baseline and follow-up. In total, 25 focus groups were undertaken with mixed gender pupils (*n* = 120) aged 11–14 years.

### Data collection

All interviews with teachers took place at the convenience of participants within school time, normally on a one to one basis in staff rooms. Interviews were guided by a topic guide (available from the lead author) based on the objectives of the study and existing literature, and focused on questions that explored perceptions of the benefits and barriers to engagement with the intervention over time, and the potential effect of this engagement on physical, emotional, school and social functioning. Interviews lasted between 10 and 30 min. Given saturation of thematic findings across interviews, we have used extracts from the 12 teachers who participated in both baseline and follow up to illustrate key themes in the results section.

Focus groups with pupils were undertaken in convenient quiet locations near to regular PE classes, alongside the collection of quantitative data, by experienced researchers (Duika Burges Watson (PhD), Jean Adams (PhD), Liane Azevedo (PhD), and Catherine Haighton (PhD)), at baseline and at 12 month follow up. Focus groups, consisting of 3–6 pupils, were led by a topic guide (available from the lead author) that were based on the objectives of the study and existing literature, and focused on questions that explored pupils expectations of the dance mats, experience and access to exergames outside school and at follow up their reflections of, and use of dance mats in school. Focus groups lasted between 10 and 15 min.

#### Data analysis

All interviews and focus groups were audio recorded and transcribed verbatim. Analysis involved listening to recordings and checking against transcripts for accuracy and to develop high level themes in NVivo by the lead qualitative researcher (DBW). A framework approach [21] was employed to map sub themes within the meta-framework informed by studies of science and technology (STS) [22, 23]. As dance mats are a technological and social intervention, transcripts were analysed using a sociology of translation approach – making no a priori distinction between the technology and the social context. This involved more than a focus on context but also consideration of the ‘problematisation’ of how stakeholders are brought into the process and how they use the technologies. A more detailed explanation of the steps involved - inter-definition of actors, inter-assessment, enrolment, obligatory passage points and mobilization – can be found elsewhere [22, 23].

## Results

Anonymised profiles of the twelve teachers who took part in both baseline and follow up interviews are shown in Table 1.

**Table 1:** Profiles of the 12 teachers with data at both time points

ID	Gender	Role
S1a	Male	Faculty lead on sport
S1b; S2b; S5a; S5b; S3a	Female	PE teachers
S1c; S3a; S3b;	Female	Dance teachers
S1 d; S2a;	Male	PE teachers
S4a	Male	School sports lead

S(no.) = school

The headings and sub-headings listed below reflect the themes and sub-themes derived from our data, quotes are used to illustrate key themes.

### Expectations of use: teachers

At baseline, teachers reported very different expectations about the mats and how they could best be employed. Decisions about use involved a trade-off between perceived appropriateness as an activity for PE classes and other school based PA opportunities, and meeting the skill needs and requirements of the curriculum and sport. Views on the appropriateness of the dance mats as an activity were initially split on the basis of ideas about the mats as play, game, sport or exercise. For most respondents, the mats were not considered ‘sport’ but rather as a game or playful activity. One of the teachers had the following view:

*“No computer games would ever take the place of sport, not my idea of proper sport, but at least it is some form of physical activity. They have some potential, but I prefer the old fashioned face to face confrontation, competition and participation, but that’s a personal preference, not whether they are effective or not”.* S2a

One teacher described looking beyond ‘tradition’, suggesting some pupils were unlikely, and unwilling, to engage in sports such as football, and that dance mats could be employed as an alternative ‘game’ with inter-collegiate competition.

*“[We have to] look beyond the traditional sports of rugby, football, hockey, netball…most [professional] people with their eyes open can see that that works for quite a lot of kids, but it doesn’t work for every kid”.* S1d

At the same time, another teacher felt that dance mats were more about engaging those disengaged from physical activity in any form.

*“I thought it would be a good way to sort of engage some of the students who wouldn’t engage quite so readily in kind of traditional sports”.* S1 b

The dance mats were, at least in expectation, considered as likely to produce engagement as they were similar to technologies such as *Wii Fit* and *Play Station* that pupils were familiar with. Several teachers noted the high use of video games in the region, with two teachers suggesting a relationship between deprivation and higher than national average video game play.

*“In this neighbourhood they may not have carpet on the floor but they’ll have every console”.* S1a

The dance mats were thus viewed less as *sport*, compared to activities such as football and cricket, and more as a video game or means to encourage PA where no ‘skills’ could be taught other than as some suggested, an introduction to dance or means of engagement in physical activity. At best, dance mats were viewed as a non-traditional game that could increase activity levels for those for whom sport ‘doesn’t work’, particularly for girls who it was generally assumed would enjoy them the most.

*“Yeah they are good, they serve as an alternative and they get not so active people involved. They have some great benefits. But they are just one of the pieces of equipment and I think they’ll only appeal to the girls that enjoy dancing”.* S3a

A key concern for teachers was whether the dance mats would be ‘effective’ in encouraging skills development, and if they would be of pedagogic value in the time limited constraints of the curriculum. It was anticipated that beyond the 6 week pilot in PE sessions, they were most likely to be used in after school and lunch time activities, particularly during ‘enrichment programmes’ (after-school programmes based on a topic that will lead to new in-depth learning).

*“My only concern is quite how you use it in a lesson and progress and what they’re getting from it. Say you had it as a unit of work, what are they going to get from that unit of work? …we’re going to experiment and try and use them in curriculum time as well as after school and evaluate it, see how it goes…”* S5b

While teachers expressed concerns about high obesity rates and low levels of PA amongst pupils, most argued that increasing PA was not the responsibility of the school, and therefore had few expectations about the value of the dance mats for PA, other than as ‘incidental’ to the curriculum.

*“It’s just not feasible for us to be responsible for their overall physical activity with 2 ½ hours a week available to us. We are of course concerned about obesity rates and so on, but we also have to deliver a sports curriculum. We can introduce kids to sport to some extent, but parents just have to take the lead on keeping their kids active”* S2a

Teachers felt that school PE concentrated on skills training and the introduction of a range of new sporting activities from which pupils could develop independently and beyond the confines of the curriculum. Moreover, they emphasised the limited time and space available for PA within PE classes and other scheduled activities such as dance, in school time.

*“It’s difficult, because as a PE teacher I would want the children to be active as much as possible in an hour’s lesson; however, if they’re teaching skills, the activity level becomes less. But until they have the skills they can’t keep the activity going. So you have to forgo some activity to get the skill level up”* S5a

### Expectations of use: pupils

Feedback from pupils revealed a high level of frustration at the lack of time and suitable spaces available for PA, particularly during lunch hours.

*“Lunch times! I want to be active but you don’t have time. If you don’t eat your dinner in a certain time there is no time to go outside“.* Girl S1

Pupils talked about long queue times for lunch, the lack of space for play relative to their previous (primary) schools and the poor facilities. Follow up questions about what they meant by facilities was often expressed in terms of embarrassment at public exercise; and for girls, the personal sense of exposure involved in getting changed for, or being seen ‘doing’, exercise in school.

*“Yeah, it was easier at primary school because there was more space and time, and you could get changed without people looking at you”*. Girl S2

For pupils across all of the focus groups there was frequent discussion and comparison of the technological sophistication of the dance mats and comparison with exergames they had at home, particularly *Wii Fit* and *Playstation*. Almost all the pupils reported having at least one movement based exergame at home, and many had experienced arcade based dance mats - they anticipated the school systems would be commensurate. One described it as replacing ‘thumbs’ with ‘feet’ – suggesting that dance mats were more video game than dance activity. Both boys and girls expressed excitement at the prospect of the mats being used in the school.

*“You just use your feet instead of your thumbs”*. Boy S3

*“I guess they’ll be like the arcade dance mats? Yeah!”.* Girl S4

At the same time some boys did regard the mats as a dance activity most suitable for girls, but more frequently, the mats were read as a computer game that they would happily engage with on those terms.

*“It’s mainly the lasses who do it but some of the lads will do it too. You just do it, like, for the fun. Like no one’s going to take the mick, it’s valid, like it’s a computer game and better than just standing around and just talking to your mates or something”.* Boy S2

### Use and non-use of mats at follow-up

A year on, the use of the mats was highly variable between and schools, and between pupils even within the same school. All schools maintained records of the use of the mats by the individuals in the linked quantitative study [14]. Use across the schools varied from only being used for enrichment activities after school, to full incorporation into 6 week ‘blocks’ of PE lessons. The highest use school (S3), had pupils who had never used the mats through to some who had used them more than 20 times. Another school (S1) was singled out by teachers in several of the others as an ‘exemplar’ of good practice. This school had organised an after school inter-collegiate dance mat based competition and had attempted to establish inter-school competition until, under a separate programme, the transport needed to move the mats was no longer available. Some teachers had successfully integrated the use of the mats within specific programmes, when the weather was not suitable for traditional outdoor activities, for dance and boxing classes, and for key ‘harder to reach groups’ - most notably amongst older girls. While isolated examples suggest the mats were used frequently in some settings, the majority of respondents felt the use of mats was limited in routine PE classes because of other contextual issues, curriculum pressures, and because of technological and human failures. Each of these elements is considered below.

### Contextual issues

The School Sports Partnership (SSP) was a government scheme introduced in 2000 to increase participation amongst 5–16 year old school children to at least 2 h of ‘high quality PE and school sport’ per week [24]. For teachers, the target of 2 h of high quality PE was frequently spoken about as unrealistic in curriculum time. However part-way through our evaluation (March 2011) there was a decline in funding to the SSP associated with a change in government (May 2010). This decline was considered by participants as a major ‘loss’, resulting in decreased opportunities for activities that engage the ‘non-football types’ and for after school and enrichment activities to improve PA. Moreover, the focus of policy on particular sports was noted as a feature of the then incumbent government’s push for an ‘Olympic legacy’ [25].

*“We used them* [dance mats] *more initially than we do now because we have the national curriculum to follow and all the Olympic legacy stuff … so um … what we try to do now is fit them in to lunch times and what’s left of the after school enrichment programme”.* S3b

Most schools had, until the change of government, timetabled extensive ‘enrichment’ programmes that provided students with time for optional activities both within non-class time and after school. Dance mats were used as one of many options, and in most settings students were encouraged to try as many activities as possible to improve their skill levels. When the dance mats were used however, they were very popular and teachers were frequently surprised at their success, even with sometimes ‘apprehensive’ boys.

*“Originally the girls were much better, and er, the lads were a little bit more apprehensive…but you do notice the improvement in foot work co-ordination”* S1a

Teachers noted the increased reaction times, and multi-sensory skills employed by pupils using the mats as well as being surprised by how girls appeared to lose their normal inhibitions about being seen exercising, even if others looked on. Unfortunately many of these enrichment initiatives were cut midway through the evaluation period.

*“It’s like they’ve got 5 sets of eyeballs…well how can you do that and watch the arrows at the same time?”* S3

*“It’s like they are in a bubble; when they are on the mats they just don’t notice whose looking on and they go for it”*. S1

### Curriculum pressures

Given the priorities within the PE curriculum to include both skills and exercise; most teachers felt the balance of these priorities precluded the use of the mats beyond occasional sessions. Moreover, pressures on the curriculum were also seen to have increased with a change in government. The views of teachers were that, particularly in the first two years of secondary school, effort should be directed towards encouraging pupils to try as many new activities as possible and to increase skill levels. In general, teachers continued to argue that PA was only possible outside PE lessons not within.

*“…to be honest we’ve not put it* [dance mats] *on the curriculum, because I felt that there’s only so much you can do in terms of learning”.* S2b

### Technological/human failures

All teachers reported problems with using the dance mat technology and most felt they were not using the mats to their full potential. Not all had received the initial training and many found updating the systems and adding new music difficult.

*“They are not always easy, for example one day one of the mats seemed to have reset itself, and I couldn’t get the mats to work, both mats had the same ID no. or something. There hasn’t been as much support in how to use them as I thought there was going to be”*. S5b

Noted also in focus groups with pupils, the technology lost its interest if they became ‘bored’ by the repetition of particular songs and having the latest ‘hit’ tracks was for some, a key motivation.

*“You can do different songs and its great when you get Lady Gaga and stuff, just…it’s better not doing the same old songs all the time”.* Girl S2

While ongoing access to technical support from the dance mats manufacturers had been part of the initial purchase agreement negotiated by the local authority, teachers either did not access it, or found it hard to access. Problems with short battery life and access to funding for new batteries could be seen either as technological or institutional failure. One teacher felt this reflected a lack of institutional support and a budgeting problem. In contrast, for another teacher these problems reflected a simple failure of the technology itself.

*“its our fault we needed to know more about updating and changing the songs that kind of thing and keeping them up to date and working… from my enrichment budget I’ve spent, god, easily over £200 in batteries”.* S4

*“I think they’ve been very successful, but they were let down by the battery life”.* S3

### Measuring success

How teachers measured the success of the programme varied, but there was little expectation that the dance mats would impact on children’s PA levels within regular PE classes. Teachers were surprised at the idea ‘one’ technology would even be considered in this way.

*“Well I can’t see how they were ever going to impact on overall physical activity in the region, we simply have too many things going on. How could you possibly measure that anyway?”* S1a

Rather the ‘success’ of the dance mat intervention was as an adjunct to existing activities. In particular, the dance mats helped to engage those disengaged from PA and provide a playful alternative means of PA.

*“I couldn’t say that its had a significant impact overall, but I can say that for certain children that may not like traditional sports they have engaged more with the dance mats…it’s been a really good starter”.* S3b

Innovation in how the mats were used was very much at the behest of individual teachers. One, who had used the mats to increase ‘reaction time’ for boys learning boxing, reported that ‘the boys loved the dance mats’.

*“The boys loved the dance mats. All the boxers loved it. We love using them but they are out of action at the minute”.* S1

Another justified the use of the mats in terms of improvements in mathematics skills and multi-tasking (S3).

*“they know who’s coming first and what percentage they’ve got and what’s happening on the sides. It’s beneficial* [for mathematic issues] *for sure.”* S3

Several teachers reported successful use of the dance mats with older girls disengaged from PA, and where there was more time available to them to use the mats.

*“Some of the older students here, the dance mats have been a permanent option as part of a PE programme. So they’ve had five choices and dance mats have been one of those choices and it’s been the same group of girls and they’ve been the less abled, in the sense of sporty”* S2

Both boys and girls said they enjoyed using the mats, when they were used. Some became ‘bored’ after the initial ‘wow’ factor had worn off, particularly if the music had not been updated to the latest tunes.

*“The dance mats, they are alright like, but after a while like once you’ve used them quite a lot the novelty wears off – but they are still fun like. It’s like a laugh as well, for people who aren’t as good as everyone else it’s a good laugh”* Boy S5

Girls and boys described ‘getting sweaty’ and teachers confirmed that the activity was high intensity.

*“Initially I didn’t believe that people would expend a lot of energy. But from what I’ve seen of people on dance mats, they work an awful lot harder than a) they would imagine they do, and b) I think anybody saying – you are going on a dance mat- would imagine them to do. I’ve watched some people on the dance mats and they are absolutely shattered at the end, really. Much more exhausted from 30 min on a dance mat than they would be from 60 min in a PE lesson”*. S1b

## Discussion

### Summary of results

This qualitative component of a mixed method study offers critical insight into some of the contextual factors that explain the successful and unsuccessful elements of the dance mat programme.

The success of the dance mats were in encouraging engagement in PA amongst harder to reach groups, particularly girls disengaged from ‘traditional sports’ like football, but also with particular groups of boys – for example those interested in improving skills for boxing. Moreover, girls appeared less inhibited in using the mats than in other activities. As others have recognised, screen based exergames can lead to a feeling of ‘immersion’, that is, “a metaphorical term derived from the physical experience of being submerged in water…the sensation of being surrounded by a completely other reality, as different as water is from air, that takes over all of our attention, our whole perceptual apparatus…” [26]. As Pasch argues [27], movement based gaming may encourage engagement because it is immersive and feels a safe, playful environment that reduces anxiety. There were other benefits that had not been anticipated by teachers or students that included mathematical skills, reaction times and co-ordination. Even though there were no expectations about the dance mats value for PA, and despite limited time and opportunity to use them, the intensity of PA during use surprised some teachers.

However, the dance mats were not employed to their full capacity, not least because of an already full, and changing, agenda for school sport. Simple technological failures such as the lack of batteries to run the mats, and lack of support to keep dance tunes updated, meant that they were not used as much as they could have been, and pupils became bored by the repetition. The combination of a change in the overall focus of school sports and the patchy use of mats across the intervention area support the lack of demonstrable findings of an overall impact on PA from our linked quantitative study [14]. But even more, the place of PA within the curriculum, both within and without timetabled PE classes, was not as central to the teachers as might have been anticipated by the project steering group. PA – despite its benefits to health and well-being, was not always, if rarely, the imperative for PE lessons – sport was.

The technologies themselves ‘created’ opportunities in some settings and in others were not even considered as having the possibility of use. As Nancy and Bingham [28] argue, it is all too easy in studies of technological innovation to focus on the innovation itself without considering where it ‘fits’ in an already complex world. The analysis we employed involved the abandonment of a priori distinction between the natural/physical and social world. That is, we did not assume the technology to ‘be’ outside of the social context in which it was employed. Employing this lens, it is possible to see how the technologies and people needed to align in particular ways and that the dance mats did not get used with promotion of PA in the school population in mind.

### Strengths and limitations

In comparison to previous studies, key strengths of our approach were the longer term focus on implementation of exergaming, the theoretical foundation informing our analysis, and that it was conducted alongside a quantitative analysis. We included both teachers’ and pupils’ perspectives and considered their expectations and views of the dance mats. This longitudinal mixed method approach may be of value in other settings of similar complexity in teasing out some overlooked elements of how innovative technologies are employed in practice.

It should be noted that there was a high drop-out of number of interviewed teachers from baseline (*n* = 20) to follow-up (*n* = 12) because of redundancy due to changes in government scheme which might have underrepresented the views of teachers who were initially exposed to the dance mat intervention. In fact the whole study took place during a period of political change effecting school sports. This could be viewed as a limitation because the findings are not generalizable to other settings; however, there were themes and issues that were not related to the politics of use, but rather fundamental practical issues that could have been easily overcome. Throughout this qualitative study there was a strong message of a mismatch between what PE actually is (sports, motor skills, and cooperative behavior) and what the intervention project instigators thought it was (PA). Moreover, we did not fully explore how the national curriculum for school sports extended beyond regular PE classes and the priorities placed on increased PA in other settings such as dance classes and ‘enrichment’ programmes. In retrospect, formally capturing this by completing in depth interviews with the project instigators and better exploring how the curriculum was interpreted by school sports leads would have helped us to explore this mismatch further.

### Interpretation of results

Under the agreement issued at the time of the implementation, all schools participating in the programme were only required to utilize the mats in the first term in PE classes. Moreover, the programme was disrupted by a change of government and funding cuts to school sports less than 12 months into the programme. The top level management of the programme weakened and many of the initiators and SSP leads lost employment. The qualitative results suggest that beyond the initial 6 week timetabled programme, use became patchy.

Part of the ‘problem’ with the dance mats for some teachers was their views of the mats’ appropriateness given their views of what the mats were ‘for’ (e.g. play, game or sport). As Shaw [29] pg. 20 sets out; play can be typified as “behaviour for the purposes of fun and enjoyment with no utilitarian or abstract goal in mind”. Play becomes game when competition is involved. Game is “any form of playful competition whose outcome is determined by physical skill, strategy or chance”. For example when not keeping score it is play, when keeping score it becomes a game. Sport is “institutionalised competitive play involving physical skill, strategy and chance”. Sport involves a higher degree of organisation with things such as governing bodies and leagues.

Dance mats could be, and were, used in different ways; for sport (eg. intercollegiate competition), play (for fun) and/or as a game (because physical skill may be involved). Our findings suggest that it is how teachers viewed and used the mats that determined whether they were for play, game or sport, not the technology itself. One implication of this is that pitching PA innovations such as dance mats as competitive ‘sport’ to PE teachers may be a better way to achieve buy in for their use by these teachers, than letting teachers decide what the innovations are for. However, for pupils, it is apparent that the game/play element of dance mats is what provides motivation to participate. This is in line with the evidence base cited earlier that the transitional drop off in PA warrants more focus on non-competitive activities supported by peers [1, 5]. A further implication is that to ensure use of exergames, more consideration of their value to different actors is required.

In addition, the suitability of the dance mats as play, game or sport in different settings needs to be considered. Teachers seemed to consider play as something for extra-curricular (lunch, after school) time, whereas game was appropriate for enrichment programmes, but that it was sport that should go into PE lessons. Unfortunately the dance mats were often seen as a diversion or at best enabled some quite targeted skill development.

## Conclusions

Our evidence suggests that there is a need for a broader view of the value and effectiveness of dance mat exergame systems especially in a school based setting. Our linked quantitative study suggested the dance mats were not particularly effective in increasing PA, but the qualitative results show that the dance mats were not used routinely enough to warrant an examination of their impact on PA. The use of the dance mats could have been improved if technological issues had been better addressed (eg batteries, updates) and if teachers had a better understanding of their use in different settings and value beyond ‘sport’.

This highlights the importance of using mixed methods to evaluate complex interventions in real world settings [16]. However, if we truly want to understand the value of dance mats in improving PA, a critical element is that the mats are specifically employed for that purpose. In our research setting, this was not the case. There was a clear tension between sport and PA given different stakeholders’ views of their respective places across and beyond the school curriculum. Those planning any intervention for promoting PA in schools need to understand the distinction between PA and PE and the tensions that exist when PE teachers are put under pressure to ensure pupils do more PA – particularly within PE classes.

## Abbreviations

BMI, body mass index; NHS, National Health Service; PA, physical activity; PE, physical education; SSP, School Sports Partnership; STS, studies of science and technology

## Additional file

Additional file 1: The effect of dance mat exergaming systems on physical activity and health – related outcomes in secondary schools: results from a natural experiment. (PDF 389 kb)
